# High b Value (2,000 s/mm^2^) Diffusion-Weighted Magnetic Resonance Imaging in Prostate Cancer at 3 Tesla: Comparison with 1,000 s/mm^2^ for Tumor Conspicuity and Discrimination of Aggressiveness

**DOI:** 10.1371/journal.pone.0096619

**Published:** 2014-05-06

**Authors:** Tsutomu Tamada, Naoki Kanomata, Teruki Sone, Yoshimasa Jo, Yoshiyuki Miyaji, Hiroki Higashi, Akira Yamamoto, Katsuyoshi Ito

**Affiliations:** 1 Department of Radiology, Kawasaki Medical School, Kurashiki city, Okayama, Japan; 2 Department of Pathology, Kawasaki Medical School, Kurashiki city, Okayama, Japan; 3 Department of Urology, Kawasaki Medical School, Kurashiki city, Okayama, Japan; Eberhard-Karls University, Germany

## Abstract

**Objective:**

The objective of our study was to investigate tumor conspicuity and the discrimination potential for tumor aggressiveness on diffusion-weighted magnetic resonance imaging (DW-MRI) with high b value at 3-T.

**Materials and Methods:**

The institutional review board approved this study and waived the requirement for informed consent. A total of 50 patients with prostate cancer (69 cancer foci; 48 in the PZ, 20 in the TZ, and one in whole prostate) who underwent multiparametric prostate MRI including DW-MRI (b values: 0, 1000 s/mm^2^ and 0, 2000 s/mm^2^) on a 3-T system were included. Lesion conspicuity score (LCS) using visual assessment (1 = invisible for surrounding normal site; 2 = slightly high intensity; 3 = moderately high; and 4 = very high) and tumor-normal signal intensity ratio (TNR) were assessed, and apparent diffusion coefficient (ADC, ×10^−3^ mm^2^/s) of the tumor regions and normal regions were measured.

**Results:**

Mean LCS and TNR at 0, 2000 s/mm^2^ was significantly higher than those at 0, 1000 s/mm^2^ (*p*<0.001 for both). In addition, ADC at both 0, 1000 and 0, 2000 s/mm^2^ was found to distinguish intermediate or high risk cancer with Gleason score ≥7 from low risk cancer with Gleason score ≤6 (*p*<0.001 for both). Furthermore, ADC of tumor regions correlated with Gleason score at both 0, 1000 s/mm^2^ (ρ = −0.602; *p*<0.001) and 0, 2000 s/mm^2^ (ρ = −0.645; *p*<0.001).

**Conclusions:**

For tumor conspicuity and characterization of prostate cancer on DW-MRI of 3-T MRI, b = 0, 2000 s/mm^2^ is more useful than b = 0, 1000 s/mm^2^.

## Introduction

More than 240,000 men in the United States had a diagnosis of prostate cancer in 2012, and the disease is the second most common cause of cancer-related deaths for men in most Western countries [1], [2]. In addition, the incidence of prostate cancer in Japan continues to increase as the proportion of elderly constituting the general population increases and due to the westernization of dietary habits; therefore, morbidity and mortality rates are expected to increase to several times their present rates [3], [4]. Accordingly, early detection and accurate characterization of prostate cancer is essential to reduce mortality rates.

Multiparametric prostate magnetic resonance imaging (MRI) is a powerful tool for detection and localization of prostate cancer that combines various functional MR techniques with conventional morphological T2-weighted imaging (T2WI) [5]. In particular, diffusion-weighted MRI (DW-MRI), which is a functional MR technique that does not use contrast agents, is noninvasive and can easily acquire images with fast image acquisition and high contrast resolution [6]. Image characteristics of DW-MRI are reflected by differences in the rates of water molecule movement in different biologic tissues, and this movement is inversely correlated with tissue cellularity [6]. Therefore, restriction of the water molecule movement, as assessed by DW-MRI, might be a useful indicator of prostate tumor aggressiveness and malignant potential [5], [6]. In addition, DW-MRI can evaluate differences in water molecule diffusion by qualitative visual assessment using relative signal intensity and also by quantitative assessment by calculating the apparent diffusion coefficient (ADC) [5], [6]. Although the ADC represents capillary perfusion and diffusion characteristics, use of a large b value can reduce the influence of capillary perfusion [7], [8]. In addition, a large b value can effectively suppress the T2 shine-through effect, which is otherwise depicted as high signal intensity on DW-MR images independent of diffusion restriction [9], [10]. Thus, a higher b value can result in stronger diffusion-weighting and greater suppression of signal intensity in benign tissue without a conspicuous decrease in the water molecule diffusion, thereby increasing image contrast between the cancer lesion and benign tissue [11]. However, a high b value also induces a decrease in the signal to noise ratios (SNR) and an increase in the susceptibility artifact and image distortion [11]. Therefore, prostate DW-MRI with a standard 1.5-Tesla (1.5-T) MR scanner is typically conducted using a b value between 500 and 1000 s/mm^2^ [12]. Alternatively, the use of higher b values, such as 2000 s/mm^2^ might be adapted for the detection and characterization of prostate cancer, since a 3-T MR scanner results in a 2-fold increase in SNR compared to a 1.5-T MR scanner. Few studies have focused on the utility of a high b value for DW-MRI including ADC at 3-T for the diagnosis of prostate cancer [11], [13]–[18]. Regarding the tumor detection ability and lesion conspicuity of prostate cancer, Rosenkrantz et al. [11], Ueno et al. [17] and Ohgiya et al. [18] reported that b = 2000 s/mm^2^, as compared to b = 1000 s/mm^2^, was superior. Conversely, Koo et al. [14] and Kim et al. [16], reported that b = 1000 s/mm^2^, as compared to b = 2000 s/mm^2^, was superior. In addition, regarding evaluation of tumor aggressiveness by ADC, contrary to expectations Kitajima et al. [13] reported that although ADC for both b = 2000 s/mm^2^ and b = 1000 s/mm^2^ showed negative significant correlation with Gleason score, the correlation coefficient was weak. To clarify the significance of a high b value for the tumor detection, lesion conspicuity and evaluation of tumor aggressiveness, further investigation is necessary.

Therefore, the goal of this study was to investigate tumor conspicuity and the discrimination potential for tumor aggressiveness on DW-MRI, including an ADC acquired with a high b value at 3-T.

## Materials and Methods

### Patient characteristics

This retrospective study was approved by the institutional review board of the Kawasaki Medical School, and the requirement for obtaining informed consent from patients was waived. All clinical investigations were conducted in accordance with the Declaration of Helsinki. Patient records/information was anonymized prior to analysis.

A total of 57 consecutive patients with biopsy-proved prostate cancer underwent MR imaging of the prostate with a 3-T system between April 2012 and March 2013 at our institution. MR examination was performed within 3 months before TRUS-guided biopsy in all patients. Seven patients were excluded based on the following criteria: incomplete MR examination (n = 2), malignant micro-lesions smaller than 1 mm (n = 3), and post-hormonal therapy (n = 2). Thus, 50 male patients (mean age, 70 years; age range, 53–85 years) were included in this study. Median PSA level was 11.96 ng/mL (range, 2.35–648 ng/mL). The median time between MR imaging and biopsy was 20 days (range, 1–67 days). No patient had undergone any treatment for cancer at the time of the prostate MR examination. Of these patients, 17 underwent radical prostatectomy within 4 months after prostate MR examination.

A urologist with 16 years of experience in prostate biopsy obtained a total of 12 specimens (eight from the PZ, four from the TZ) from each patient for all patients during TRUS-guided systematic prostate biopsy. Biopsy sites included base right, middle right, apex right, far lateral right, base left, middle left, apex left, and far lateral left regions in the PZ, and bilateral ventral and dorsal regions in the TZ.

### MR imaging

After intramuscular administration of glucagon or buscopan to decrease intestinal peristalsis, MR imaging was performed under fasting conditions using a 3-T MR scanner (Vantage Titan 3-T; Toshiba Medical Systems, Tochigi, Japan; maximum gradient amplitude, 30 mT/m; maximum slew rate, 203 mT/m/s) with a 16-channel phased array coil (Atlas SPEEDER Body combined with Atlas SPEEDER Spine; Toshiba Medical Systems) for signal reception.

In our institution, the protocols for prostate MR imaging consisted of axial T1-weighted fast spin-echo (FSE) imaging, axial and coronal T2-weighted FSE imaging, axial DW-MRI, and axial dynamic contrast-enhanced MR imaging (DCE-MRI). The technical parameters of all MRI pulse sequences, including DW-MRI, are listed in Table 1.

**Table 1:** Sequence parameters for prostate MR protocol

Sequence	T1-weighted FSE	T2-weighted FSE	T2-weighted FSE	DW-MRI (Single-shot SE EPI)	DCE-MRI (3D GRE)
Plane	Axial	Axial	Coronal	Axial	Axial
TR (msec)	870	4300	5000	7100	5.5
TE (msec)	12	120	120	95	2.5
ETL	4	19	19	NA	NA
Flip angle (degrees)	NA	NA	NA	90	15
Matrix Size	320×256	384×384	512×192	128×256	320×192
Field of View (cm)	24×24	24×24	24×24	36×36	35×35
No. of Acquisition	1	4	1	3	1
Slice Thickness (mm)	3	3	3	5	3
Interslice Gap (mm)	0.3	0.3	0.3	1	0
Parallel Imaging Factor	2	1.8	NA	2.5	2
Scan Time	2 min 4 s	6 min 28 s	3 min 10 s	3 min 12 s	3 min (30 s×6 phases)

DW-MRI was acquired with motion-probing gradient (MPG) pulses applied sequentially along 3 orthogonal orientations with two kinds of b value sets (0 and 1000 s/mm^2^ or 0 and 2000 s/mm^2^). (TR - repetition time, TE - echo time, ETL - echo train length, FSE - fast spin-echo, DW-MRI - diffusion weighted MR imaging, DCE-MRI - dynamic contrast-enhanced MR imaging, GRE - gradient-echo, NA - not applicable).

Axial DW-MRI was performed using a multi-section spin-echo single-shot EPI sequence. For ADC calculation, DW-MRI was acquired with motion-probing gradient (MPG) pulses applied sequentially along three orthogonal orientations following acquisition at a b value of 0 s/mm^2^. DW-MRI was acquired with two kinds of b value sets (0 and 1000 s/mm^2^, and 0 and 2000 s/mm^2^). All other parameters for these DW images were kept constant. ADC maps were reconstructed by calculating ADC in each pixel of each slice, and the ADC values were calculated for a pair of b values: 0 and 1000 s/mm^2^, or 0 and 2000 s/mm^2^. These images were obtained within an acquisition time of 3 min 12 s.

DCE-MRI was performed using a three-dimensional T1-weighted gradient-echo sequence with fat-suppression technique (Quick 3D). Data acquisition for DCE-MRI began simultaneously with initiation of intravenous injection of gadopentetate dimeglumine (Magnevist; Bayer Schering Pharma, Osaka, Japan) of 0.1 mmol/kg body weight at a rate of 3 mL/s via a power injector, followed by a 40-ml saline flush at the same rate of Gd-DTPA injection. Multiphase DCE images were obtained every 30 s for 3 min (six phases) without breath-holding.

### Histopathologic analysis

In this study, the reference standard for tumor localization of prostate cancer was determined using the step-section histologic slices in 17 patients who underwent radical prostatectomy following prostate biopsy and in the US-guided biopsy specimen in 33 patients who underwent other therapies (such as radiotherapy).

Prostatectomy specimens were sliced from the apex to base at 4- to 6-mm intervals in a plane perpendicular to the prostatic urethra. The distal portion of the apex and the proximal portion of base were amputated and were sliced sagittally to assess the resection margin. All slices were processed uniformly and submitted entirely. After routine hematoxylin-eosin staining, a pathologist with 20 years of experience in genitourinary pathology evaluated all the pathologic specimens and outlined the location of the tumor or tumors on the photographs for each slice [19]. Each tumor was graded according to the 2005 International Society of Urological Pathology Modified Gleason Grading System [20].

For the evaluation of US-guided biopsy specimen, the prostate gland was divided into eight regions on MR images according to the sites of prostate biopsy; the base right in PZ, middle right and far lateral right in PZ, apex right in PZ, ventral and dorsal right in TZ, base left in PZ, middle left and far lateral left in PZ, apex left in PZ, and ventral and dorsal left in TZ were designated as base right, middle right, apex right, TZ right, base left, middle left, apex left, and TZ left, respectively [21], [22].

To distinguish the PZ from the TZ, reviewers mainly used morphological T2-weighted MR images that provide anatomical information. Landmarks used to distinguish the PZ from the TZ were the urethra and the surgical pseudocapsule. In the PZ, sections through the bladder neck and proximal prostatic urethra were considered as base, whereas the prostatic apex was defined by the doughnut-shaped appearance of the distal prostatic urethra. The remainder of the PZ was considered as middle [23].

### Image interpretation and data analysis

Image evaluation was performed using a PACS viewer (Rapidey Core; Toshiba Medical Systems). At first, the prostate MR images and the pathological reports of prostate biopsy or the whole-mount pathologic step-section slices of all patients were reviewed to identify prostate cancer foci that were subsequently targeted for the image analysis in consensus by a radiologist (the role of a study coordinator) with 14 years of experience in prostate MR imaging and a pathologist with 20 years of experience in genitourinary pathology. Cancer foci were selected if they showed positive finding with 5 mm or more on at least one of the three MR imaging sequences, including T2WI, DW-MRI and DCE-MRI. A tumor site was considered to match histological findings if the tumor depicted on the image was present in the same region of the prostate indicated in the pathology report on the US-guided biopsy specimen or prostatectomy specimen. For each MR imaging sequence, lesions fulfilling the following criteria were regarded as prostate cancer [24]: *a)* on T2WI, an area of homogeneous low signal intensity with mass effect in the PZ, an area of circumscribed round-shaped localized signal hypointensity in the PZ, or an area in the TZ with homogeneous signal hypointensity, ill-defined margins, and lack of capsule, with or without a lenticular shape and invasion of the anterior fibromuscular stroma; *b)* on DWI and ADC maps, an area with focal high signal intensity on DWI and with focal low signal intensity on the ADC map relative to the background prostatic parenchyma; or *c)* on DCE-MRI, an area with focal early enhancement until the third phase (60 s after contrast media administration). T2-weighted images were referred to when interpreting images from DWI and DCE-MRI, so as to confirm anatomical positions after the assessment of each image. In addition, reviewers also recorded the Gleason score of prostate cancer in selected lesions from the pathologic report on the US-guided biopsy specimen or radical prostatectomy. The Gleason score of all selected lesions was determined by the same pathologist to avoid inter-observer variability.

For qualitative DW-MRI assessments of the selected lesions, two other radiologists with 10 years of experience in abdominal radiology reviewed the DW-MRI set consisting of a DW-MR image and an ADC map of each lesion that were acquired with b values of 0, 1000 s/mm^2^ or 0, 2000 s/mm^2^ to evaluate the lesion conspicuity by consensus. To minimize recognition bias, the radiologists were randomly assigned one of two kinds of DW-MRI sets (0, 1000 s/mm^2^ and 0, 2000 s/mm) of each lesion with each set separated by at least 3 weeks by a study coordinator and were blinded to the b value that was used. The radiologists recorded the lesion conspicuity score (LCS) relative to the surrounding benign prostatic tissue in each MR image using the following 4-point grading scale: 1 =  invisible for surrounding benign site (isointense lesions); 2 =  slightly high (questionable, poorly visible abnormal hyperintense lesion); 3 =  moderately high (hyperintense lesion with reasonable conspicuity); and 4 =  very high intensity (hyperintense lesion with excellent conspicuity) in DW images, and 1 =  invisible for surrounding benign site (isointense lesions); 2 =  slightly low (questionable, poorly visible abnormal hypointense lesion); 3 =  moderately low (hypointense nodular lesion with reasonable conspicuity); and 4 =  very low intensity (hypointense nodular lesion with excellent conspicuity) in ADC maps.

Next, the same two radiologists who performed the qualitative analysis reviewed the DW-MRI set consisting of a DW-MR image with 0, 1000 s/mm^2^, an ADC map with 0, 1000 s/mm^2^, a DW-MR image with 0, 2000 s/mm^2^ and an ADC map with 0, 2000 s/mm^2^ of each lesion to quantitatively evaluate the lesion conspicuity of the tumor regions and to measure the ADC value of the tumor regions and normal regions by consensus. The tumor-normal signal intensity ratio (TNR) of each tumor region in the DW-MR image was calculated from SI values of tumor regions (SI tumor) and surrounding benign prostatic parenchymal zone (PZ or TZ) corresponding to the tumor lesion (SI prostate) as SI tumor/SI prostate. ADC measurements were performed using the region of interest (ROI) placement technique for ADC maps on the PACS monitor. The SI and ADC were measured by means of a ROI placed by the same two radiologists. Each ROI was a circle or oval and was chosen to be as large as possible. To minimize the position gap of ROI placements, the four MR images from each tumor region were evaluated in parallel on a PACS monitor, and the ROIs of tumor regions and surrounding benign prostatic parenchyma in one of these four MR images was extrapolated to the other three MR images of each subject using “copy and paste” and visual fine adjustment. For SI and ADC measurements of the tumor region, the ROIs were placed throughout the lesion. For SI and ADC measurements of surrounding prostatic parenchyma, the ROI was placed at a location that was considered normal prostatic parenchyma near the lesion on the basis of histopathological findings from patients who underwent prostatectomy or from biopsy result and multiparametric MR findings in patients who underwent biopsy only. When setting the ROI in the benign parenchyma, great care was taken to exclude the urethra, periprostatic venous plexus and neurovascular bundle to reduce errors in SI measurements and ADC calculations.

### Statistical analysis

Statistical analyses were performed using SPSS software (version 19.0; SPSS, Chicago, IL). Wilcoxon signed rank test was used to determine significant differences in the LCS of lesion in DW-MR images, the LCS of lesion in ADC maps and the TNR of lesion in DW-MR images between b values of 0 and 1000 s/mm^2^ and b values of 0 and 2000 s/mm^2^, and in the ADC values at tumor regions or normal regions between b values of 0 and 1000 s/mm^2^ and b values of 0 and 2000 s/mm^2^. The ADC values at b values of 0 and 1000 s/mm^2^ or b values of 0 and 2000 s/mm^2^ between tumor regions and normal regions or tumor regions with Gleason score ≥7 (intermediate or high risk prostate cancer) and tumor regions with Gleason score ≤6 (low risk prostate cancer) were compared by using a Mann–Whitney U test. Relationships between mean ADC values in tumor regions at b values of 0 and 1000 s/mm^2^ or b values of 0 and 2000 s/mm^2^ and tumor Gleason score were assessed using the Spearman rank correlation coefficient (ρ). Furthermore, for discrimination between tumor regions with Gleason score ≥7 (intermediate or high risk prostate cancer) and tumor regions with Gleason score ≤6 (low risk prostate cancer), ADC cutoff values were determined so that the sum of sensitivity and specificity was maximized. Differences in sensitivity and specificity between b values of 0 and 1000 s/mm^2^ and b values of 0 and 2000 s/mm^2^ were tested using the McNemar test. Microsoft Excel 2010 software was used to determine significant differences in the correlation coefficient (ρ) between b values of 0 and 1000 s/mm^2^ and b values of 0 and 2000 s/mm^2^. A *p* value <0.05 was taken to indicate a statistically significant difference.

## Results

### Prostate pathology

TRUS-guided prostate biopsy in 33 patients and radical prostatectomy in 17 patients demonstrated a total of 69 prostate cancers. These tumors were located in the PZ in 48 lesions (70%), the TZ in 20 lesions (28%), and both PZ and TZ in one lesion (2%). The PZ lesions included eight lesions in the base, 24 lesions in the middle, and 16 lesions in the apex. The median Gleason tumor score of these lesions was 7 (range, 5–9), and the Gleason grade classifications for all 69 prostate specimens were as follows: one (1.5%) was grade 3+2; 12 (17%) were grade 3+3; 12 (17%) were grade 3+4; 18 (26%) were grade 4+3; 17 (25%) were grade 4+4; eight (12%) were grade 4+5; and one (1.5%) was grade 5+4.

### Tumor conspicuity

**Figure 1:**
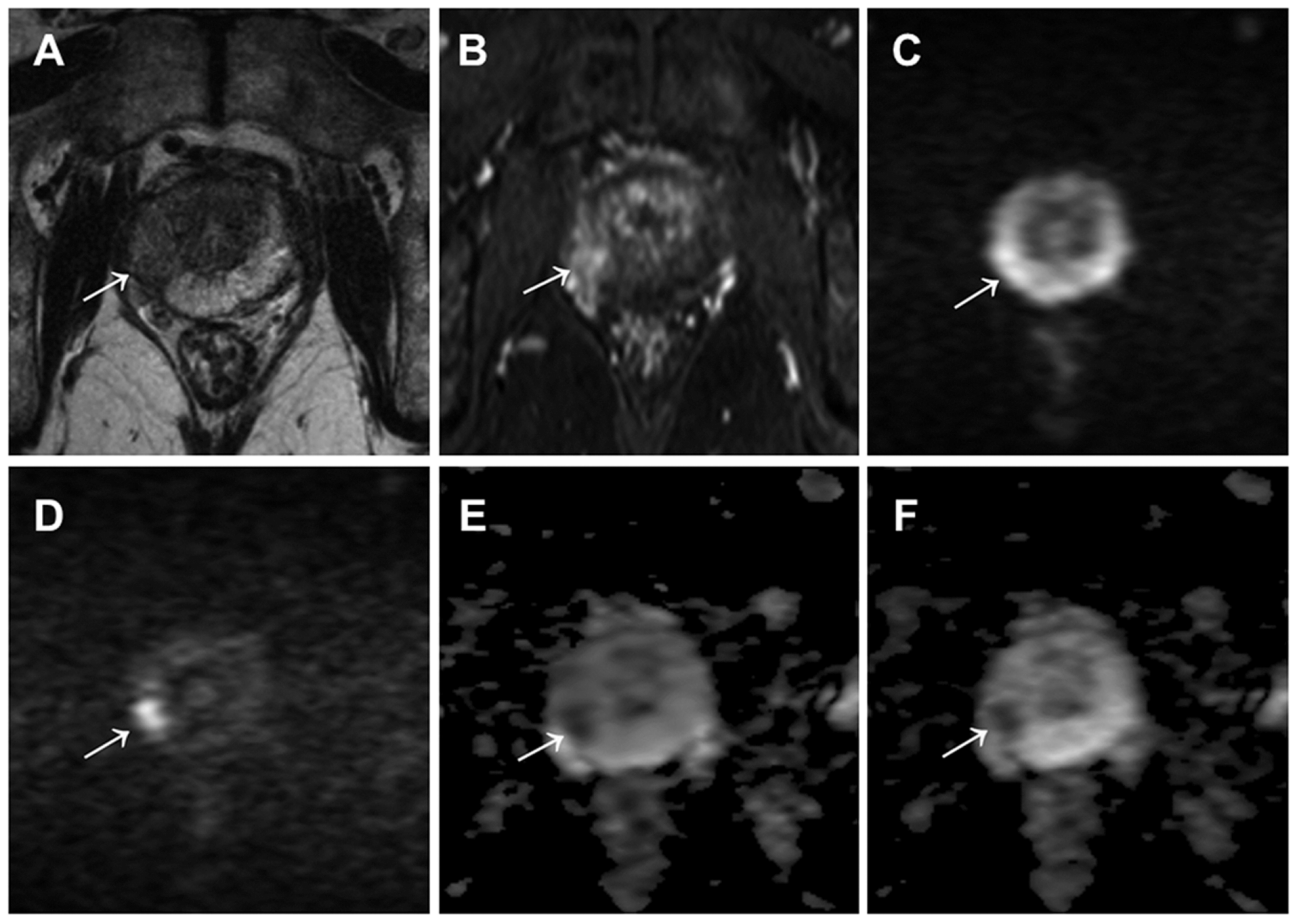
A 77-year-old male with prostate cancer (PSA level of 7.45 ng/mL, Gleason score of 4+3) in the middle right region in the peripheral zone. Cancer lesion is shown as a homogeneous hypointense lesion with mass effect on T2-weighted imaging (arrow) (A) and focal early enhancement on dynamic contrast-enhanced MR imaging (arrow) (B). The lesion with a focal hyperintensity is depicted clearly in the DW image with b values of 0 and 2000 s/mm^2^ (arrow) (D), as compared with the DW image with b values of 0 and 1000 s/mm^2^ (arrow) (C). However, the lesion conspicuity with a focal hypointensity in the ADC map with b values of 0 and 2000 s/mm^2^ (arrow) (F) was equivalent to that of ADC map with b values of 0 and 1000 s/mm^2^ (arrow) (E).

In DW-MR images, mean LCS and TNR was significantly higher at b values of 0, 2000 s/mm^2^ than at b values of 0, 1000 s/mm^2^ (3.20±0.98 vs. 2.26±1.24 and 1.98±0.83 vs. 1.36±0.54, respectively; *p*<0.001 for both). On the other hand, mean LCS in ADC map was equivalent when comparing b values of 0, 2000 s/mm^2^ (3.19±0.96) and b values of 0, 1000 s/mm^2^ (3.26±0.93) (*p* = 0.132) (Figure 1) (Table 2).

**Table 2:** Qualitative and quantitative analyses of two b values protocols for lesion conspicuity of prostate cancer in the DW-MR image and ADC map.

	b = 0 and 1000 s/mm^2^	b = 0 and 2000 s/mm^2^	*p* values
LCS			
DW-MR image	2.26±1.24*	3.20±0.98	<0.001
ADC map	3.26±0.93	3.19±0.96	0.124
TNR			
DW-MR image	1.36±0.54*	1.98±0.83	<0.001

Data are given as mean±standard deviation. (LCS - lesion conspicuity score, TNR - tumor-normal signal intensity ratio).

^*^Significant differences between b = 0 and 1000 s/mm^2^ and b = 0 and 2000 s/mm.

### ADC values in the regions of prostate cancer versus benign prostatic parenchyma

**Figure 2:**
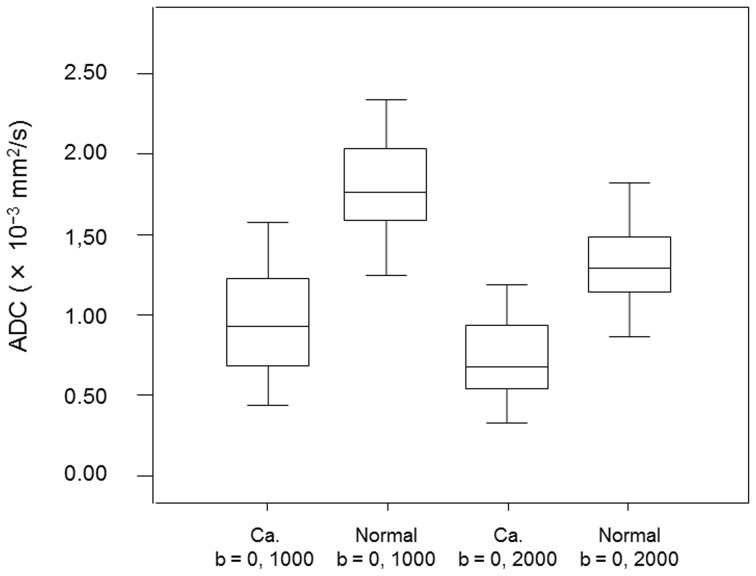
Box and whisker plots show ADC values of prostate cancer lesions (Ca.) and benign prostatic parenchyma (Normal) obtained with b values of 0 and 1000 s/mm^2^ or b values of 0 and 2000 s/mm^2^. Central horizontal line  =  median, top of box  =  75th percentile, bottom of box  =  25th percentile, vertical line  =  data range.

Mean ADC at 0, 1000 and 0, 2000 s/mm^2^ was significantly lower in tumor regions than that in normal regions (0.96±0.32×10^−3^ vs. 1.79±0.28 and 0.73±0.22 vs. 1.32±0.24, respectively; *p*<0.001 for both) (Figure 2).

### ADC values of prostate cancer and benign prostatic parenchyma at b values of 0, 2000 s/mm^2^ versus 0, 1000 s/mm^2^

In both the tumor and normal regions, mean ADC values were significantly lower for b values of 0, 2000 s/mm^2^ than for b values of 0, 1000 s/mm^2^ (0.73±0.22 ×10^−3^ vs. 0.96±0.32 and 1.32±0.24 vs. 1.79±0.28, respectively; *p*<0.001 for both) (Figure 2).

### ADC value in intermediate or high risk prostate cancer versus low risk prostate cancer

**Figure 3:**
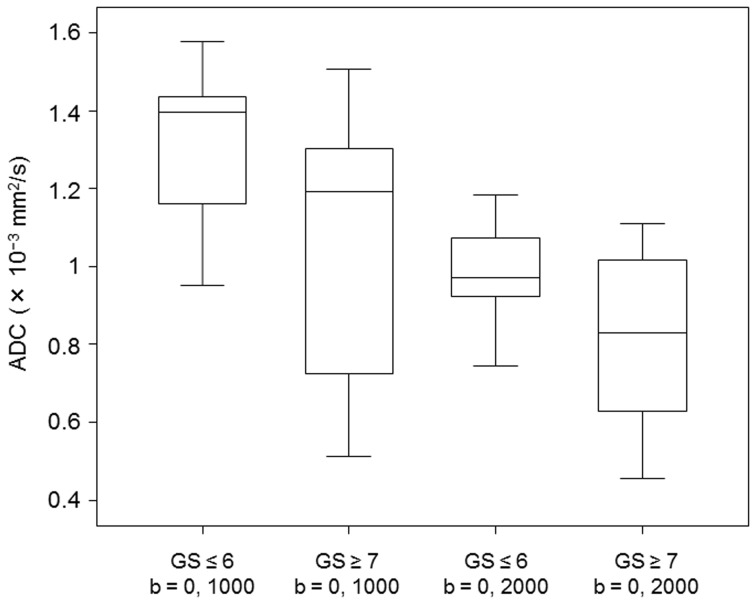
Box and whisker plots show ADC values of low risk prostate cancer lesions with Gleason score ≤6 and intermediate or high risk prostate cancer lesions with Gleason score ≥7 obtained with b values of 0 and 1000 s/mm^2^ or b values of 0 and 2000 s/mm^2^. Central horizontal line  =  median, top of box  =  75th percentile, bottom of box  =  25th percentile, vertical line  =  data range.

ADC values of b values of 0, 1000 s/mm^2^ and b values of 0, 2000 s/mm^2^ were found to distinguish intermediate or high risk tumors with Gleason score ≥7 from low risk tumors with Gleason score ≤6 (0.88±0.30×10^−3^ vs. 1.30±0.20 and 0.67±0.20 vs. 0.97±0.13, respectively; *p*<0.001 for both). However, for b values of 0, 1000 s/mm^2^ and 0, 2000 s/mm^2^, there was the overlap of the ADC values between intermediate or high risk cancer lesions and low risk cancer lesions (Figure 3). In terms of discrimination between intermediate or high risk cancer lesions and low risk cancer lesions using ADC values, a cutoff value of 1.16×10^−3^ in b values of 0, 1000 s/mm^2^ resulted in a sensitivity of 77% and a specificity of 77%, and a cutoff value of 0.92×10^−3^ in b values of 0, 2000 s/mm^2^ resulted in a sensitivity of 82% and a specificity of 77%. Although, no significant differences were observed in the sensitivity of both groups (*p* = 0.250), these data suggest that b values of 0, 2000 s/mm^2^ are associated with high sensitivity for a detection of intermediate or high risk prostate cancer.

### Correlation between ADC values and tumor aggressiveness

**Figure 4:**
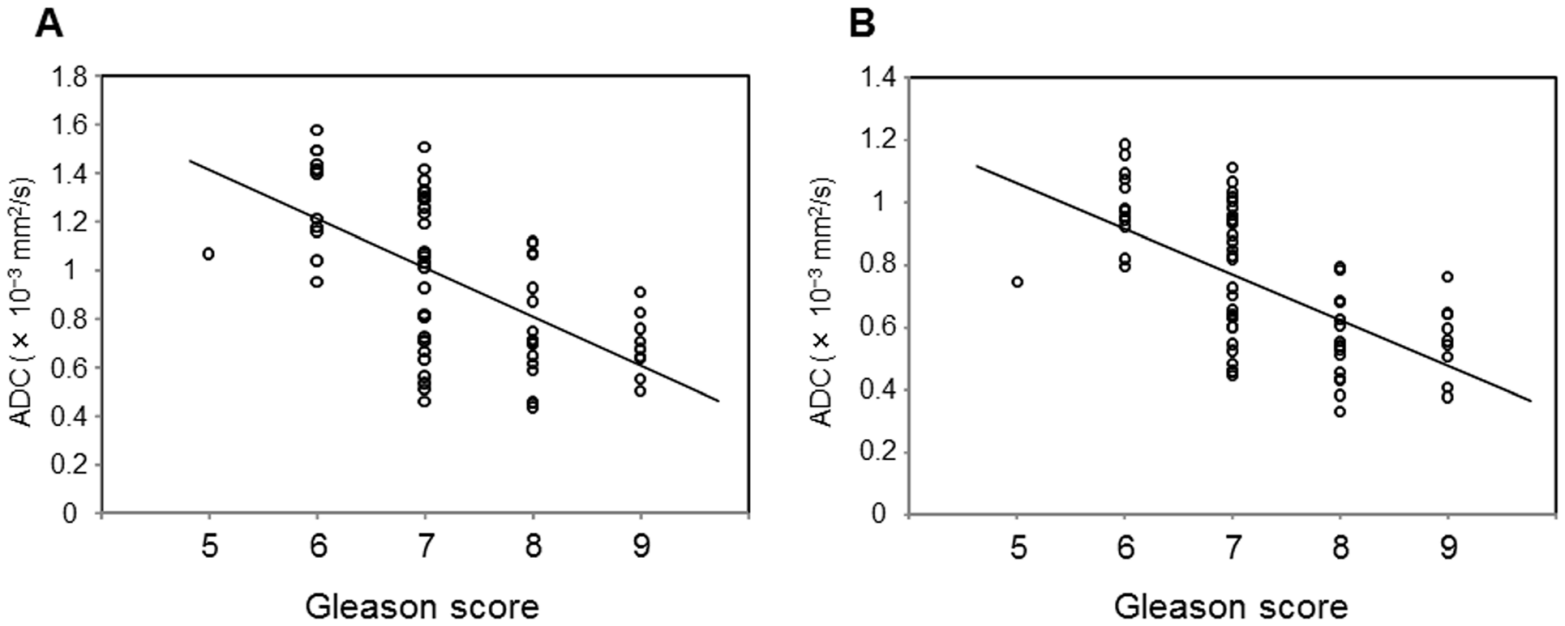
Scatterplots showing the relationship between ADC values in prostate cancer and tumor Gleason scores. (A) b values of 0 and 1000 s/mm^2^. (B) b values of 0 and 2000 s/mm^2^. ADC values in b values of 0 and 1000 s/mm^2^ and b values of 0 and 2000 s/mm^2^ each negatively correlated with tumor Gleason score (ρ = –0.602, *p*<0.001; ρ = –0.645, *p*<0.001, respectively).

ADC values of tumor regions correlated with tumor Gleason score at 0, 1000 s/mm^2^ (ρ = –0.602; *p*<0.001) and 0, 2000 s/mm^2^ (ρ = –0.645; *p*<0.001). Although, no significant differences were observed in the correlation coefficient (ρ) of both groups (*p*  = 0.149), these data suggest that ADC with b values of 0, 2000 s/mm^2^ might be superior to ADC with b values of 0, 1000 s/mm^2^ for discrimination of tumor aggressiveness (Figure 4).

## Discussion

The present qualitative and quantitative analyses of prostate cancer lesion conspicuity showed that DW-MR images at b values of 0, 2000 s/mm^2^ was significantly higher than those at b values of 0, 1000 s/mm^2^, although lesion conspicuity of ADC maps between b values of 0, 1000 s/mm^2^ and b values of 0, 2000 s/mm^2^ was equivalent. In the clinical setting, radiologists generally detect lesions with high signal intensity on DW-MR images and confirm a decreased ADC of the lesion on ADC maps. Therefore, such high lesion conspicuity in DW-MR images at b values of 0, 2000 s/mm^2^ is very useful for detection of prostate cancer. Signal intensity is suppressed at surrounding normal regions, and signal intensity at cancer regions is increased, thereby resulting in good conspicuity of prostate cancer when performing DW-MR imaging using b values of 0, 2000 s/mm^2^ relative to b values of 0, 1000 s/mm^2^. This may be because higher b values provide increased diffusion-weighting and diminished T2 shine-through [6]. Furthermore, a decrease in SNR which is one of the disadvantages of higher b values would be overcome by using a 3-T MR system rather than a 1.5-T system. The present results regarding lesion conspicuity on DW-MRI are consistent with findings by Rosenkrantz et al. [11], who strongly recommended the use of DW-MRI with high b values for the detection of prostate cancer. Furthermore, high b value DW images might help inexperienced radiologists to diagnose prostate cancer based on the high signal intensities on DW-MRI.

The present study also demonstrated that there was no difference in lesion conspicuity on ADC maps when comparing b values of 0, 1000 s/mm^2^ and b values of 0, 2000 s/mm^2^. This may be because the ADC map is a pure reflection of diffusion contrast when compared with DW-MRI with the superimposed T2 weighting and diffusion-weighting. Accordingly, when using b values of 0, 2000 s/mm^2^, ADC maps and DW-MRI will play a complementary role for improvement of the tumor detectability of prostate cancer each other.

ADC values were significantly lower for cancer regions than normal regions when using b values of either 0, 1000 s/mm^2^ or 0, 2000 s/mm^2^. Moreover, as shown in Fig. 2, there was little overlap between groups in both b values, suggesting that ADC using either b value was similarly useful for detection of prostate cancer. Other researchers have suggested that the overlap between ADC values in normal regions and cancer regions cannot be ignored, and limitations in differentiating normal tissue from malignant disease have been reported [5], [25]. Benign prostatic lesions, such as prostatic hyperplasia (particularly stromal hyperplasia) in the TZ and chronic prostatitis in the PZ, often shows decreased ADC values [25]–[27]. Some studies have included these benign prostatic lesions in the normal region. Meanwhile, in a study using b values of 0, 1000 s/mm^2^ at a 3-T [28] with ADC cut-off values of 1.67×10^−3^ mm^2^/s for PZ and 1.61×10^−3^ mm^2^/s for TZ, the sensitivity and specificity for tumor detection was 94% and 91%, respectively, for the PZ, and was 90% and 84%, respectively, for the TZ. These data suggest that the ADC value at high field strength was useful for the detection of prostate cancer.

On the other hand, when using high b values for DW-MRI at 3-T, radiologists should recognize that the ADC values in cancer regions and normal regions tend to decrease along with the increase in b value, as shown in Fig. 2 and in previous studies [13]. The reason could be explained by the non-Gaussian kurtosis effect which deviates from a monoexponential signal decay and adequately represents tissue microstructure [29].

The current gold standard for assessment of the aggressiveness of prostate cancer is the Gleason score obtained from prostate biopsy specimens or obtained from radical prostatectomy specimens. Evaluation of tumor aggressiveness using the ADC value is a useful clinical application of DW-MRI, as it is non-invasive when compared with prostate biopsy. Several studies using a 1.5-T or a 3-T system have reported a significant negative correlation between the Gleason score and ADC in cancer regions (combined TZ cancer and PZ cancer) obtained using various b values [30]–[33]. The correlation coefficient for the relationship between Gleason score and ADC in cancer regions was −0.24 to −0.376 with a 1.5-T system using b values of 0, 600 to 1500 s/mm^2^ [30], [31] and was −0.38 to −0.55 with a 3-T system using b values of 0, 500 to 800 s/mm^2^ [32], [33]. In the present study using a 3-T system, the correlation coefficient was −0.602 using b values of 0, 1000 s/mm^2^ and was −0.645 using b values of 0, 2000 s/mm^2^. Accordingly, the correlation coefficient tended to indicate a stronger relationship between these variables when using a 3-T system than when using a 1.5-T system and when using a higher b value than with standard b values. Proton diffusion properties of water in high b value DW-MRI with increased S/N, diffusion-weighting and diminished T2 shine-through may more strongly reflect the cellular density and structural changes of gland stroma in prostate cancer, which are important for the determination of tumor aggressiveness, when compared with standard b value DW-MRI. On the other hand, in a study by Kitajima et al. using the ADC with b values of 0, 2000 s/mm^2^ at 3-T, the correlation coefficients were −0.323 to −0.341 [13]. This discrepancy in comparison to our results may be due to the differences in patient proportion for each tumor Gleason score and in Gleason grade classification between both studies. The ratio of patients in Gleason score ≤6 (low risk), Gleason score 7 (intermediate risk) and Gleason score ≥8 (high risk) were 18.5%, 43% and 38.5% in our study and were 13%, 73% and 14% respectively, in their study. Furthermore, they divided Gleason score 7 into 3+4 and 4+3, and Gleason score 9 into 4+5 and 5+4. Accordingly, the heterogeneity of the patient’s distribution for tumor Gleason grades and the excessive classification for Gleason score in their study might be disadvantageous for the correlative analysis between Gleason score and ADC in tumor lesions. However, even in the desirable results such as high correlation between GS and ADC obtained with high b values in our study, because of the overlap in the ADC values between Gleason score grades, use of ADC values to predict the Gleason score may be difficult.

The present study investigated the ability of ADC values to discriminate between low risk cancers and intermediate or high risk cancers using ADC value and showed that ADC cut-off values of 1.16×10^−3 ^mm^2^/s in b values of 0, 1000 s/mm^2^ were associated with a sensitivity and specificity of 77% and 77%, respectively. By contrast, with ADC cut-off values of 0.92×10^−3 ^mm^2^/s in b values of 0, 2000 s/mm^2^, the sensitivity and specificity were 82% and 77%, respectively, suggesting that high b values were better at detecting intermediate or high risk prostate cancer when compared with standard b values. Previous studies have also reported that the ADC can discriminate between low risk cancers (Gleason score ≤6) and intermediate or high risk cancers (Gleason score ≥7) [30], [34], [35]. Therefore, in patients with prostate cancer, the ADC value may serve as a prognostic indicator.

This study has several limitations. First, the patient population was relatively small. Second, MR image findings in 66% (33/50 patients) of study subjects were not compared with the results of step-section histologic mapping using the whole prostate following radical prostatectomy. This might result in an underestimation of the Gleason score of prostatic tumors evaluated by systematic TRUS-guided prostate biopsy specimens [36]–[38]. Third, for the acquisition of DW-MRI, TE in b values (95 msec) of 0, 1000 s/mm^2^ was set as the same value as in b values of 0, 2000 s/mm^2^ for comparison. Therefore, an unnecessarily long TE for the b values of 0, 1000 (generally, about 70 msec) compared to b values of 0, 2000 s/mm^2^ might be given and as a result, a substantial reduction of the potential SNR in the b values of 0, 1000 which has influence on the lesion conspicuity in DW-MR images might occur. Fourth, this study analyzed PZ cancers and TZ cancers as one group. However, it is not realistic to evaluate the ADC value of TZ cancers and PZ cancers separately in the clinical setting, and therefore, analyzing PZ tumors and TZ tumors as one group might more accurately reflect clinical practice. Finally, this study was performed in a retrospective manner. Further prospective investigations with larger patient populations in whom there is a direct correlation between histologic section findings and tumor conspicuity and between radical prostatectomy GS and the ADC value are needed to clarify the clinical value of high b value DW-MRI for prostate cancer detection and assessment of tumor aggressiveness.

## Conclusions

The use of high b values (0, 2000 s/mm^2^) for prostate cancer detection and assessment of tumor aggressiveness on 3-T DW-MRI was associated with increased lesion conspicuity and the ability to differentiate between low risk and intermediate to high risk cancer in a noninvasive manner when compared with the use of standard b values of 0, 1000 s/mm^2^.
